# Comparison of different auxiliary techniques used during root canal filling removal in terms of the amount of apically extruded debris: In vitro study

**DOI:** 10.1371/journal.pone.0323807

**Published:** 2025-05-15

**Authors:** Ismail Uzun, Kevser Şenel, Rawan Alqawasmi

**Affiliations:** 1 Department of Endodontics, Faculty of Dentistry, Ondokuz Mayis University, Samsun, Turkey; 2 Department of Endodontics, Ordu Central Dental Hospital, Ordu, Turkey; 3 Department of Endodontics, Faculty of Dentistry, Arab American University, Jenin, Palestine; University of Puthisastra, CAMBODIA

## Abstract

**Background:**

One of the main challenges in endodontic retreatment is managing apical debris extrusion, which can influence both healing and patient comfort. Different retreatment methods result in varying levels of extrusion. This study aims to quantitatively compare the extent of apical debris extrusion caused by different auxiliary techniques during the extraction of root canal fillings in mandibular molars, to help guide the selection of an optimal endodontic approach.

**Materials and methods:**

Sixty mandibular molar teeth scheduled for extraction due to periodontal reasons, such as advanced bone loss and periodontal disease, which rendered them non-restorable, were collected. All extractions were performed with prior informed consent from the patients, ensuring adherence to ethical standards. Preparation was performed with the Protaper Next file system, focusing on X3 files according to the manufacturer’s guidelines. Teeth were filled using the lateral compaction technique with AH Plus sealer and stored at 100% humidity at 37°C for two weeks for the sealant to set. The samples were divided into four groups (n = 15 each): Protaper Universal Retreatment (PTUR), Ultrasonic + PTUR, Orange Oil + PTUR, and System B + PTUR, with PTUR procedural steps followed to attain working length. During preparation, 15 ml of distilled water was used, followed by 1 ml of distilled water for debris collection post-procedure. The debris was incubated at 68°C for five days to evaporate the water, and tube weights were recorded and compared statistically across groups.

**Results:**

The study assessed debris extrusion during endodontic retreatment. The PTUR group showed the least extrusion (average 1.1 mg, SD ± 1.05 mg), indicating a more controlled approach. The Ultrasonic + PTUR group exhibited higher extrusion (average 4.2 mg, SD ± 2.12 mg), reflecting a more invasive technique with a greater potential for debris extrusion. The Orange Oil + PTUR group displayed moderate extrusion levels (average 2.5 mg SD ± 1.46 mg), reflecting the solvent’s effect. The System B + PTUR group had the highest extrusion (average 4.3 mg, SD ± 1.87 mg), indicating it as the method associated with the greatest debris displacement. Statistically significant differences were found between the PTUR group and the other groups (P < 0.05). Additionally, a significant difference was observed between the Orange Oil + PTUR group and both the Ultrasonic + PTUR and System B + PTUR groups (P < 0.05). No significant difference was noted between the System B + PTUR and Ultrasonic + PTUR groups (P > 0.05).

**Conclusion:**

The study concludes that auxiliary methods used during root canal filling removal significantly impact the degree of apical debris extrusion, with some methods leading to greater extrusion than others.

## Introduction

Nonsurgical endodontic retreatment is the primary approach for eliminating or reducing microbial infection when initial root canal therapy fails [1]. The main objective of this procedure is to remove the root canal filling material, allowing renewed access to the apical foramen and facilitating further cleaning, shaping, and refilling of the root canals [2]. Research has shown that during retreatment, irritants such as filling material residues, necrotic pulp tissue, bacteria, or irrigants may be extruded into the periradicular tissues [3]. Clinically, these apically extruded materials have been linked to postoperative discomfort, including inflammation, postoperative pain and even failure of apical healing [4,5]. Multiple studies indicate that the amount of apical debris extrusion varies based on the preparation technique and the design of root canal instruments used [6,7].

Several techniques are commonly employed to remove root canal filling material, including the use of solvents, heat-producing devices, hot hand instruments, mechanical methods, and ultrasonic devices [8–10]. It is widely observed that manual instrumentation tends to result in greater apical extrusion compared to engine-driven rotary preparation [11]. According to Huang et al. [3], the ideal retreatment technique should aim to remove as much of the root canal filling material as possible while minimizing apical extrusion.

However, despite the available research, there is a lack of comprehensive comparative analyses examining how auxiliary techniques like ultrasonic activation, thermal techniques (System B), and solvents such as orange oil influence apical debris extrusion when used alongside standard protocols like PTUR. Ultrasonic activation has been shown to enhance the removal of filling materials but may simultaneously increase apical extrusion due to the agitation of debris toward the apex [12]. Orange oil, as a safer alternative to solvents like xylol, effectively softens gutta-percha but its precise role in modulating debris extrusion remains under investigation [13]. System B is widely used in endodontic practice for the warm vertical compaction of gutta-percha due to its ability to enhance the adaptation of filling material to the canal walls [14]. However, its impact on apical debris extrusion during retreatment procedures remains underexplored. By including System B in our study, we aimed to investigate whether its thermal effects during filling removal influence the amount of apically extruded debris, thus addressing a gap in the current literatüre [15].

In endodontic procedures, the mechanical removal of root canal filling materials frequently involves chemical solvents specifically formulated to dissolve gutta-percha and various root canal sealers [16]. Extensive research has been conducted to identify effective solvents for gutta-percha removal, leading to substantial advancements in this field [17,18]. However, despite their high efficacy in dissolving gutta-percha, solvents such as xylol and chloroform pose significant health risks, being potentially carcinogenic and neurotoxic [19]. These risks have raised concerns regarding their effects on periapical tissues [20].

In the search for safer alternatives, orange oil has emerged as a promising solvent [21]. It has been effectively used to dissolve zinc oxide and eugenol-based sealers. Pécora et al. [22] highlighted that orange oil can soften gutta-percha cones during endodontic retreatment, showing results comparable to xylol while offering a safer alternative. Recent studies confirm that orange oil demonstrates similar efficacy to chloroform in dissolving obturated materials while presenting fewer health risks [13]. Derived from the peel of sweet orange (*Citrus aurantium*), orange oil provides an accessible and efficient solution for rapid root canal opening, particularly in zinc-oxide cement root fillings, regardless of the presence of gutta-percha cones. Its superiority over potentially toxic solvents underscores its suitability as an excellent option for softening and dissolving gutta-percha and for use with eugenol zinc-oxide cement. The dissolution efficacy of organic solvents like orange oil on gutta-percha further reinforces its potential as a safer alternative in endodontic treatments [23].

Passive Ultrasonic Activation (PUA) is commonly employed as an auxiliary technique to enhance the removal of gutta-percha and sealers from root canal systems. PUA involves the agitation or activation of a solution using ultrasound energy without concurrent irrigation [24]. This method is particularly effective in removing hard pastes, cements, and sealers such as glass ionomer cement and is frequently used during retreatment procedures [25].

In the context of gutta-percha removal, ultrasonic files activated without irrigation produce frictional heat, which plasticizes gutta-percha, making it easier to remove. However, this technique has certain limitations [26]. Thermoplasticized gutta-percha may accumulate along the canal walls, leading to significant debris [27]. Additionally, ultrasonic files are generally effective only in the straight portions of the canal. Despite these limitations, ultrasonically activated files have been found to be an efficient method for removing the bulk of the material [26].

In many endodontically treated teeth, a post is required to improve the retention of the coronal restoration, necessitating the removal of the coronal portion of the gutta-percha using thermal methods, among other techniques [28]. However, this approach carries the risk of heat conduction to the periodontal attachment unit, potentially causing permanent damage, especially when the remaining dentin walls of the root are thin [28,29]. Although the thermal method is widely regarded as the safest and most effective means of removing gutta-percha, it is not without risk. Excessive heat can damage the dentin, cementum, periodontal ligament, and alveolar bone [30]. The transmission of heat to the surrounding tooth structure and periodontium—particularly in cases with thin dentin walls—poses a significant concern.

Endodontic retreatment is essential when initial root canal therapy fails, with various techniques employed to remove existing filling materials. Rotary systems, ultrasonic devices, and thermal techniques like System B are commonly used in clinical practice. Our literature review revealed no existing studies employing System B for endodontic retreatment or comparing it with other methods. This study is the first to evaluate System B’s effectiveness and comparative impact on apical extrusion [31].

We have now provided a clear justification for selecting the Protaper Universal Retreatment (PTUR) system. PTUR is widely used in clinical practice due to its efficiency and standardized design, which ensures consistent removal of root canal filling materials. PTUR has demonstrated superior efficiency in removing filling materials while causing minimal apical debris extrusion compared to other systems [32]. Furthermore, PTUR’s reliability is supported by its lower incidence of file fractures, making it a safer choice in clinical settings. Combining PTUR with auxiliary techniques allows for a comprehensive evaluation of their cumulative effects on apical debris extrusion [33]. The choice of retreatment technique significantly influences the extent of apical debris extrusion, which is linked to postoperative discomfort and delayed healing.

This study aims to address these gaps by systematically comparing the effects of different auxiliary methods on apical debris extrusion during root canal filling removal. By evaluating these techniques in a controlled in vitro setting, we seek to provide clearer guidance on their relative safety and efficacy, ultimately contributing to improved clinical outcomes and reduced postoperative complications in endodontic retreatment. Minimizing apical debris extrusion can reduce postoperative discomfort, inflammation, and the risk of treatment failure. By identifying auxiliary techniques that optimize debris removal while limiting extrusion, clinicians can enhance patient outcomes and improve the overall success rates of endodontic retreatments.

When we look at all these results, based on present data, it was demonstrated that irrespective of the instrumentation technique (manual or mechanical) chosen by the clinician, using auxiliary techniques during the de-obturation procedure can create disadvantages in root canal cleaning. Applying these techniques should only be considered when the previous working length cannot be reached [34].

We hypothesize that the use of different auxiliary methods for removing root canal filling material in the distal canals of lower molars will lead to varying levels of apical debris extrusion. Specifically, we expect to observe significant differences in the amount of apical extrusion between groups treated with Protaper Universal Retreatment files (PTUR), System B combined with PTUR, Solvent (orange oil) plus PTUR, and Ultrasonic plus PTUR. Our goal is to evaluate and compare the effectiveness of these auxiliary methods in minimizing apical debris extrusion during the root canal filling removal process.

## Materials and methods

### Study design and ethical considerations

This in vitro experimental study was conducted at Ondokuz Mayıs University, Faculty of Dentistry, to compare the apical debris extrusion associated with different auxiliary techniques used during root canal filling removal. A power analysis was performed to determine the sample size, aiming for an 80% confidence level with a 5% margin of error. Based on an effect size of 0.324 (from Bodrumlu et al.’s study on three techniques for root canal filling removal, p = 0.05), a minimum of 15 samples per group was required to achieve a power of 0.80, leading to the inclusion of 60 teeth in the study.

All teeth were extracted for periodontal reasons with prior informed consent from patients, following the Declaration of Helsinki. Ethical approval was granted by the Clinical Research Ethics Committee of Ondokuz Mayıs University (2022/61). Written informed consent was obtained from all participants. To ensure ethical handling of biological specimens, all extracted teeth were processed under strict biohazard disposal guidelines. The recruitment period started on March 1, 2022, and was completed on March 29, 2022.

### Tooth selection and preparation

Sixty human lower molar teeth, extracted for periodontal reasons, were selected for this study. The criteria for assessing periodontal disease included tooth mobility, severity of attachment loss, and radiographic bone loss greater than 50% [35]. Distal roots were selected due to their relatively uniform canal morphology, which allows for standardization and more consistent comparison of debris extrusion outcomes. Teeth were examined under a 6 × magnification loupe (Orascoptic Eyezoom, Middleton, WI, USA) to exclude any with external resorption, calcifications, cracks, fractures, caries, or prior root canal treatment. Only teeth with a single canal and a curvature <50° (determined using Schneider’s method) [22] were included.

The teeth were stored in distilled water before use. Endodontic access cavities were prepared with a high-speed carbide bur (Dentsply Sirona, Ballaigues, Switzerland) under water cooling. Canals with a diameter larger than ISO size 25 were excluded. All crowns were reduced to standardize the tooth length at 13 mm. The working length (WL) was determined by inserting a #10 K-file (Dentsply Maillefer) until visible at the apical foramen, then subtracting 1 mm.

### Root canal preparation and obturation

All canals were shaped using a VDW.Gold Reciproc endomotor (VDW, Munich, Germany) with ProTaper Next (Dentsply, Ballaigues, Switzerland) X1 (17.04), X2 (25.06), and X3 (30.07) files, following the manufacturer’s instructions. After each file, canals were irrigated with 2 mL of 2.5% sodium hypochlorite (NaOCl). Final irrigation included 5 mL each of 17% EDTA, 2.5% NaOCl, and distilled water.

Canals were obturated using the lateral compaction technique with gutta-percha (Dentsply Maillefer) and AH Plus sealer (Dentsply Sirona, Bensheim, Germany). The specimens were stored at 37°C in 100% humidity for two weeks to allow complete sealer setting.

### Debris collection setup

To collect apically extruded debris, an Eppendorf tube setup was used. A hole was made in the lid of each tube, allowing the tooth to be inserted up to the cementoenamel junction. A 27-gauge needle was inserted alongside the stopper to balance internal and external pressures. Each tube, containing the tooth and needle, was secured within a plastic cap to blind the operator during debris collection.

**Fig 1:**
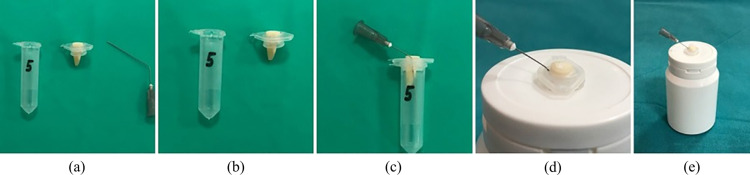
An experimental assembly engineered to quantify the volume of debris extruded apically during endodontic retreatment procedures. **(a)** The Eppendorf tube was modified by creating a hole in its cap, allowing the insertion of an extracted tooth up to the cementoenamel junction. The tapered end of the tube was left intact to serve as a collection chamber for extruded debris. **(b)** A 27-gauge needle was inserted through the cap alongside the tooth to equalize internal and external pressures, preventing pressure buildup that could alter debris extrusion. The tube was then filled with distilled water to maintain a humid environment. **(c)** The modified Eppendorf tube, now containing the secured tooth and pressure-regulating needle, was placed within a plastic cap to standardize positioning and blind the operator during debris collection. Cyanoacrylate adhesive was used to seal all connections and prevent leakage. **(d)** A close-up view showing the insertion of the irrigation needle at the working length, demonstrating the standardized irrigation protocol across all groups.(e) The final assembled setup, with the Eppendorf tube placed inside a collection vial to prevent external contamination and ensure precise debris quantification.

To prevent leakage, the Eppendorf tube connections were sealed with cyanoacrylate adhesive. Apical pressure was controlled by inserting a small needle through a puncture on the side of each tube, allowing excess pressure to vent. The apical foramen was left patent, and equal irrigation flow rates were maintained across all groups. The experimental setup is illustrated in (Fig 1).

Before retreatment, the initial weight of each tube was recorded using an analytical balance (AUW-220D, Shimadzu, Tokyo, Japan) with a precision of 10⁻⁵ g (average of three consecutive measurements).

### Retreatment procedures

The teeth were randomly assigned to four experimental groups (n = 15 per group). Canal patency was maintained by passing a #10 K-file after each instrument or technique.

1. **PTUR Group:** Protaper Universal Retreatment (PTUR) system files (D1, D2, D3) were used to remove the filling material, followed by ProTaper Next X4 (40.06) for additional shaping.
2. **System B + PTUR Group:** System B (200°C) was applied 3 mm short of the WL, then PTUR files (D1, D2, D3) were used, followed by ProTaper Next X4 (40.06).
3. **Orange Oil + PTUR Group:** Orange oil was applied for 1 minute before using PTUR files (D1, D2, D3), then ProTaper Next X4 (40.06) for final shaping.
4. **Ultrasonic + PTUR Group:** A piezoelectric ultrasonic unit (miniMaster EMS, Nyon, Switzerland) with an E7 retreatment tip was used before PTUR (D1, D2, D3), followed by ProTaper Next X4 (40.06).

A Zeiss EXTARO 300 dental microscope (10 × magnification, Carl Zeiss Meditec AG, Oberkochen, Germany) was used to assess canal cleanliness.

### Randomization and blinding

A double-blind design was implemented, where both the operator performing the retreatment procedures and the data analyst measuring the debris extrusion were blinded to group allocations. This approach ensured that both the procedural and analytical phases were free from bias.

### Debris collection and statistical analysis

After debris collection, tubes were placed in an incubator at 68°C for five days to evaporate water. The incubation setup included a calibrated laboratory oven with digital temperature controls, and temperature was monitored daily using an external digital thermometer (±0.1°C accuracy). Any fluctuations were corrected immediately.

After incubation, the final dry weight of extruded debris was calculated by subtracting the pre-experimental weight of the tubes. Statistical analysis was performed using SPSS 20 (Chicago, IL, USA). Normality testing was conducted before selecting appropriate parametric or nonparametric tests (significance level p < 0.05).

## Results

The Shapiro-Wilk test was used to assess the normality of the data before and after square root transformation. Prior to transformation, the data showed significant deviations from normality (p < 0.05). After applying the square root transformation, the Shapiro-Wilk test results indicated no significant deviations from normality (p > 0.05). This normalization justified the use of parametric tests for subsequent statistical analyses.

**Fig 2:**
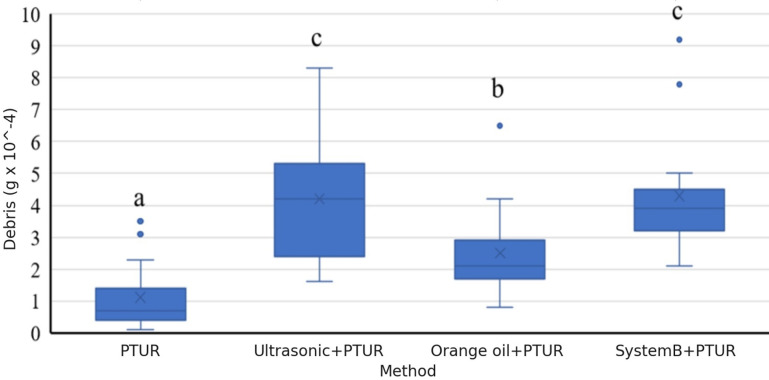
Distribution of data in different groups. (The ‘x’ mark on the graphs indicates the mean). (P < 0.05).

Following the ANOVA, Tukey’s HSD post-hoc tests were conducted for pairwise comparisons to identify specific group differences. Effect size calculations (η²) were also reported for all significant findings to provide a measure of practical significance. These additions enhance the robustness and interpretability of our statistical analysis (Fig 2).

In assessing debris removal efficacy during the root canal procedures, different irrigation protocols were utilized, and their results were quantified in terms of minimum, maximum, and mean debris weights (in units of 10^-4^grams). For simplicity, debris weights will henceforth be reported without repeating this unit.

The quantitative comparison of debris extrusion across the various endodontic retreatment methods yielded the following results:

- PTUR Group: This group exhibited the least amount of debris extrusion, with an average weight of 1.1 mg (SD ± 1.05 mg). This suggests that the PTUR method is less aggressive and provides better control over debris extrusion compared to other methods.
- Ultrasonic + PTUR Group: A higher level of debris extrusion was observed in this group, with an average weight of 4.2 mg (SD ± 2.12 mg). This finding indicates that the combination of ultrasonic activation with PTUR may lead to increased debris extrusion.
- Orange Oil + PTUR Group: The debris extrusion in this group was moderate, with an average weight of 2.5 mg (SD ± 1.46 mg). The use of orange oil as a solvent appears to have contributed to a moderate increase in debris extrusion compared to PTUR alone.
- System B + PTUR Group: This group exhibited the highest amount of debris extrusion, with an average weight of 4.3 mg (SD ± 1.87 mg). These results suggest that this method is the most aggressive in terms of debris extrusion.

The statistical analysis revealed significant differences between the groups. Specifically, the PTUR group exhibited significantly lower debris extrusion compared to the other groups (p < 0.05). However, no significant difference was found between the System B + PTUR and Ultrasonic + PTUR groups (p > 0.05), indicating that these two methods produced comparable debris extrusion levels. Additionally, the Orange Oil + PTUR group exhibited significantly different results compared to the other groups (p < 0.05), underscoring the distinct influence of the solvent.

The data and group comparison details are provided in Fig 2 and Table 1, which highlight the minimum, maximum, and mean debris weights for each group. These visual aids further clarify the distribution of debris extrusion across the different methods and underscore the significant differences observed.

**Table 1:** Minimum and maximum values of the debris extruded apically.

	Minimum m (mg)	Maximum m (mg)	Mean (mg)± SD (mg)
PTUR	0.1	0.35	0.11 ± 1.05 a
Ultrasonic+PTUR	0.16	0.83	0.42 ± 2.12 c
Orange Oil+PTUR	0.08	0.65	0.25 ± 1.46 b
SystemB+PTUR	0.21	0.92	0.43 ± 1.87 c

Statistically significant differences were found between groups (Fig 1). A statistically significant difference was found between Group 1 (PTUR) and the other groups in the removal of root canal fillings. (p < 0.05) No statistically significant difference was found between Group 2: System B + PTUR and Group 4: Ultrasonic + PTUR. (p > 0.05) A statistically significant difference was found between Group 3: Solvent (orange oil) group and the other groups. (p < 0.05).

These results highlight the varying effects of different endodontic retreatment methods on apical debris extrusion. The PTUR method demonstrated the least extrusion, suggesting its suitability as a more conservative approach, while the System B + PTUR method showed the highest extrusion, indicating its potential for greater aggressiveness.

## Discussion

Several techniques have been proposed for removing filling materials from the root canal system, including nickel-titanium rotary instruments, heated instruments, ultrasonic instruments, lasers, and adjunctive solvents [36]. This study aimed to evaluate the effectiveness of these techniques in minimizing apical debris extrusion, a factor closely associated with postoperative complications such as inflammation and delayed healing [4]. Among the methods tested, the Protaper Universal Retreatment (PTUR) system demonstrated the least amount of apical debris extrusion, making it the most conservative option compared to other auxiliary methods, such as System B + PTUR and Ultrasonic + PTUR, which exhibited higher debris extrusion levels [37]. These results suggest a potential clinical advantage in using rotary systems like PTUR to reduce debris displacement during retreatment. Previous studies have shown inconsistent results regarding the effectiveness of various retreatment techniques, highlighting the need for further research in this area [32].

Today, Nickel-Titanium (Ni-Ti) rotary instrument systems are specifically designed for retreatment procedures [38]. The PTUR file system, used in this study, includes three files (D1, D2, and D3) that effectively remove root canal fillings without engaging the canal walls. These files, with their triangular cross-section, are well-suited for removing gutta-percha [39]. Clinically, retreatment is often performed with files larger than the original master apical file to ensure adequate debridement. In this study, additional instrumentation was carried out using size 40 files due to the size 30 apical diameter in the initial preparation. To ensure accurate debris weight measurement, distilled water was used as the irrigation solution to avoid crystallization of NaOCl, which could have influenced debris weight.

The results of this study are consistent with those of previous research, including studies by Altundasar et al. [6] and Koçak et al. [7], which reported that rotary systems like PTUR produce less debris extrusion compared to more aggressive methods. In contrast, methods such as System B + PTUR and Ultrasonic + PTUR were associated with higher debris extrusion, a finding supported by Huang et al. [3], who demonstrated that ultrasonic activation and thermal techniques cause greater debris movement towards the apex due to increased agitation and thermal effects. These findings align with the literature, suggesting that the more aggressive the method, the greater the likelihood of apical debris extrusion.

The use of solvents like orange oil in this study showed moderate debris extrusion. Solvents soften the filling material, making it more adhesive to the canal walls, potentially increasing debris retention. This observation aligns with Barreto et al. [25], who found that solvents combined with ultrasonic activation can leave softened gutta-percha films on the canal walls, hindering complete debris removal. According to the findings of this study, all adjunctive methods employed, except the PTUR group, exhibited a significantly higher incidence of apically extruded debris. This phenomenon can be attributed to previous research indicating that solvents may impede the removal of root-filling material by rendering it more viscous and adhesive. This effect leads to softened gutta-percha films forming on the root canal surface, potentially penetrating irregularities or dentinal tubules [40].

Multiple studies have reported that gutta-percha remnants persist in the apical third of the root canal system even after retreatment [41,42]. Mechanical systems, even when combined with solvents, are often less effective at removing root canal fillings than manual methods [43]. This could be due to the softened gutta-percha film on the canal walls, which is influenced by the mechanical motion of the files and increased temperature. Interestingly, the PTUR files used without solvent did not exhibit the same disadvantages, suggesting that solvent use may not always be beneficial in mechanical retreatment [25,44].

In evaluating different solvents for softening gutta-percha, Oyama et al. [23] found both xylol and orange oil to be highly effective. This is crucial as gutta-percha, a rubber-like plastic material, requires softening for easier penetration and movement of manual endodontic instruments during cement removal [13]. Despite the lack of specific standards for measuring endodontic solvents on filling materials, experimental observations consider essential factors such as solvent effect time, commonly used materials, temperature, and contact surface [45].

Efficiency and similarity between xylol and orange oil suggest the potential extended clinical use of the latter due to its low toxicity [13]. However, solvents should be carefully limited to the periapical area to prevent chemical pericementitis [46]. Therefore, cautious solvent utilization, short gauge, and active endodontic files are essential to facilitate endodontic cement’s chemical–mechanical removal [8].

In the Ultrasonic + PTUR group, the lower extrusion levels were initially attributed to the larger diameter of the ultrasonic tip, which limited its apical reach. However, additional factors may also have contributed to this outcome. The frequency of ultrasonic activation likely played a role in mobilizing debris towards the apex, with higher frequencies potentially increasing debris agitation. Moreover, ultrasonic energy induces cavitation and acoustic streaming, which can enhance cleaning efficiency but may also contribute to increased apical extrusion [47]. Clinically, while ultrasonic activation is effective for improving root canal cleanliness, it necessitates careful control of activation parameters—such as frequency and duration—to minimize apical debris extrusion and reduce the risk of postoperative complications. These insights highlight the need for clinicians to balance the benefits of ultrasonic activation with its potential risks, thereby optimizing patient outcomes in endodontic retreatment [48].

While our study provides valuable insights into the effects of auxiliary techniques on apical debris extrusion, there are several avenues for future research that could further enhance our understanding of this subject. In particular, future studies could investigate the impact of different ultrasonic tip designs, including variations in diameter, shape, and material composition, on apical debris extrusion. These factors could significantly influence the dynamics of debris movement within the root canal system. Additionally, exploring novel irrigation protocols, such as negative pressure irrigation systems or the use of advanced irrigants like nanoparticle-based solutions, could provide new strategies for mitigating debris extrusion associated with auxiliary techniques. These investigations have the potential to offer deeper insights into optimizing retreatment procedures and minimizing postoperative complications, thereby contributing to improved patient outcomes in clinical practice.

From a clinical perspective, PTUR should be preferred in cases where minimizing apical debris extrusion is a priority, such as in patients with a history of periapical inflammation or heightened sensitivity to postoperative pain [33]. In contrast, auxiliary methods like ultrasonic activation or System B may be more suitable when thorough removal of filling materials is critical, such as in complex retreatments with extensive canal calcifications [49]. Furthermore, the use of orange oil as a solvent has shown potential in reducing apical debris extrusion while facilitating gutta-percha removal [50]. Clinicians should carefully balance the risk of debris extrusion with the need for effective retreatment by selecting techniques based on individual patient anatomy, clinical history, and the complexity of the retreatment case. Incorporating gentle irrigation protocols and controlling instrument parameters can further mitigate extrusion risks while ensuring effective canal cleaning.

Additionally, clinically relevant variables such as canal shape, curvature, and operator experience significantly impact apical debris extrusion. More curved and complex canal anatomies pose a higher risk of debris displacement towards the apex [51]. Operator experience also plays a crucial role, as less experienced clinicians may inadvertently apply inconsistent pressure or improper techniques, leading to increased extrusion [52]. Furthermore, the type of instrumentation system used in curved canals, such as Reciproc Blue or WaveOne Gold, has been shown to influence the extent of apical debris extrusion [53]. Future research should focus on evaluating these factors to develop standardized protocols aimed at minimizing debris extrusion while maintaining effective retreatment outcomes. This approach will help provide more personalized and practical recommendations for clinicians handling various anatomical complexities and differing levels of operator expertise.

### Study limitations

This study has several limitations. Firstly, distilled water was used as an irrigant to ensure consistent debris weight measurements, but this does not reflect clinical practice where NaOCl is typically used for its antimicrobial properties [54]. Future studies should evaluate how clinically relevant irrigants, such as NaOCl, impact debris extrusion [25].

Another limitation is the in vitro nature of the study, which does not fully account for clinical variables like patient variability, canal curvature, or operator skill. Abramovitz et al. [41] emphasized that in vivo studies provide more accurate reflections of clinical outcomes, and future research should consider these variables. Additionally, this study was conducted using mandibular molars, which may not be representative of all tooth types. Future research should explore whether similar debris extrusion patterns are observed in other tooth types, including maxillary molars, premolars, and anterior teeth.

Moreover, the restricted apical reach of the ultrasonic retreatment tip due to its larger diameter may have influenced the amount of debris extrusion observed. The tip length and diameter could limit complete debridement in the apical region, potentially affecting the outcomes.

## Conclusion

Our study demonstrated that the Protaper Universal Retreatment (PTUR) system results in the least apical debris extrusion compared to auxiliary techniques such as ultrasonic activation, System B, and the use of orange oil. While auxiliary methods enhance the removal of filling materials, they are associated with increased debris extrusion, which may lead to postoperative complications. Clinicians should consider the balance between effective retreatment and the risk of apical debris extrusion when selecting techniques, favoring PTUR in cases where minimizing extrusion is critical. These findings offer practical insights for optimizing retreatment protocols and improving patient outcomes.

## References

[1] Stabholz A, Friedman S. Endodontic retreatment--case selection and technique. Part 2: Treatment planning for retreatment. J Endod. 1988;14(12):607–14. doi: 10.1016/S0099-2399(88)80058-X 3270681

[2] de Chevigny C, Dao TT, Basrani BR, Marquis V, Farzaneh M, Abitbol S, et al. Treatment outcome in endodontics: the Toronto study--phases 3 and 4: orthograde retreatment. J Endod. 2008;34(2):131–7. doi: 10.1016/j.joen.2007.11.003 18215667

[3] Huang X, Ling J, Wei X, Gu L. Quantitative evaluation of debris extruded apically by using ProTaper Universal Tulsa rotary system in endodontic retreatment. J Endod. 2007;33(9):1102–5. doi: 10.1016/j.joen.2007.05.019 17931943

[4] Seltzer S, Naidorf IJ. Flare-ups in endodontics: I. Etiological factors. J Endod. 1985;11(11):472–8. doi: 10.1016/S0099-2399(85)80220-X 3868692

[5] Siqueira JF Jr. Microbial causes of endodontic flare-ups. Int Endod J. 2003;36(7):453–63. doi: 10.1046/j.1365-2591.2003.00671.x 12823700

[6] Altundasar E, Nagas E, Uyanik O, Serper A. Debris and irrigant extrusion potential of 2 rotary systems and irrigation needles. Oral Surg Oral Med Oral Pathol Oral Radiol Endod. 2011;112(4):e31-5. doi: 10.1016/j.tripleo.2011.03.044 21778084

[7] Koçak S, Koçak MM, Sağlam BC, Türker SA, Sağsen B, Er Ö. Apical extrusion of debris using self-adjusting file, reciprocating single-file, and 2 rotary instrumentation systems. J Endod. 2013;39(10):1278–80. doi: 10.1016/j.joen.2013.06.013 24041391

[8] Martos J, Gastal MT, Sommer L, Lund RG, Del Pino FAB, Osinaga PWR. Dissolving efficacy of organic solvents on root canal sealers. Clin Oral Investig. 2006;10(1):50–4. doi: 10.1007/s00784-005-0023-2 16317555

[9] Bodrumlu E, Er O, Kayaoglu G. Solubility of root canal sealers with different organic solvents. Oral Surg Oral Med Oral Pathol Oral Radiol Endod. 2008;106(3):e67-9. doi: 10.1016/j.tripleo.2008.05.007 18602299

[10] Martos J, Bassotto APS, González-Rodríguez MP, Ferrer-Luque CM. Dissolving efficacy of eucalyptus and orange oil, xylol and chloroform solvents on different root canal sealers. Int Endod J. 2011;44(11):1024–8. doi: 10.1111/j.1365-2591.2011.01912.x 21658077

[11] Kustarci A, Altunbas D, Akpinar KE. Comparative study of apically extruded debris using one manual and two rotary instrumentation techniques for endodontic retreatment. Journal of Dental Sciences. 2012;7(1):1–6. doi: 10.1016/j.jds.2011.10.003 24520438

[12] Keskin C, Sarıyılmaz E, Sarıyılmaz Ö. Effect of solvents on apically extruded debris and irrigant during root canal retreatment using reciprocating instruments. International Endodontic Journal. 2017;50:1084. doi: 10.1111/iej.12729 . 27917509

[13] Afrina Parvin C, Hoque T, Hasan MS, Rumon K, Hossain Ayumi A, Hossain M. Effectiveness of Chloroform and Orange Oil as Gutta-percha Solvents used in Endodontic Retreatment: An In Vitro Study. IJAMRS. 2024;4(5):447–51. doi: 10.62225/2583049x.2024.4.5.3267

[14] Zhang C, Huang W, Sun Z, Hou B. A comparison of two gutta‐percha master points consisting of different phases in filling of artificial lateral canals and depressions in the apical region of root canals when using a warm vertical compaction technique. Int Endod J. 2011;44(11):1041–6. doi: 10.1111/j.1365-2591.2011.01917.x 21906086

[15] Mahmood S, Mohmmed S. Investigation in to the efficacy of ultrasonic activation of the solvent in removal of residual obturation materials in endodontic retreatment procedures. J Res Med Dent Sci. 2020;8(5):10–5. doi: 10.5455/jrmds.2020863

[16] Schäfer E, Zandbiglari T. A comparison of the effectiveness of chloroform and eucalyptus oil in dissolving root canal sealers. Oral Surg Oral Med Oral Pathol Oral Radiol Endod. 2002;93(5):611–6. doi: 10.1067/mhn.2002.124846 12075213

[17] Wilcox LR. Endodontic retreatment with halothane versus chloroform solvent. J Endod. 1995;21(6):305–7. doi: 10.1016/S0099-2399(06)81006-X 7673838

[18] Barbosa SV, Burkard DH, Spångberg LS. Cytotoxic effects of gutta-percha solvents. J Endod. 1994;20(1):6–8. doi: 10.1016/s0099-2399(06)80018-x 8182389

[19] Schuur A, Moorer W, Wesselink P. [Solvents for the removal of gutta-percha from root canals. 2. Side effects of chloroform, halothane and xylene]. Ned Tijdschr Tandheelkd. 2004;111(8):303–6. 15384923

[20] Vajrabhaya L-O, Suwannawong SK, Kamolroongwarakul R, Pewklieng L. Cytotoxicity evaluation of gutta-percha solvents: Chloroform and GP-Solvent (limonene). Oral Surg Oral Med Oral Pathol Oral Radiol Endod. 2004;98(6):756–9. doi: 10.1016/j.tripleo.2004.05.002 15583552

[21] Mushtaq M, Masoodi A, Farooq R, Khan FY. The Dissolving Ability of Different Organic Solvents on Three Different Root Canal Sealers: In Vitro Study. Iran Endod J. 2012;7(4):198–202. 23130079 PMC3487522

[22] Pécora JD, Spanó JC, Barbin EL. In vitro study on the softening of gutta-percha cones in endodontic retreatment. Braz Dent J. 1993;4(1):43–7. . 8180484

[23] Oyama KON, Siqueira EL, dos Santos M. In vitro study of effect of solvent on root canal retreatment. Braz Dent J. 2002;13(3):208–11. doi: 10.1590/s0103-64402002000300014 12428599

[24] More A, Sumanthini M, Shenoy V. Efficacy of Rotary Retreatment Techniques Assisted with Passive Ultrasonic Activation of Resin Solvent in Removal of Gutta-percha with Epoxy Resin and MTA Based Root Canal Sealers: An In-vitro Study. J Clin Diagn Res. 2022;16(9):ZC06–10. doi: 10.7860/JCDR/2022/55339.16956 36204332

[25] Barreto MS, da Rosa RA, Santini MF, Cavenago BC, Duarte MAH, Bier CAS, et al. Efficacy of ultrasonic activation of NaOCl and orange oil in removing filling material from mesial canals of mandibular molars with and without isthmus. J Appl Oral Sci. 2016;24(1):37–44. doi: 10.1590/1678-775720150090 26200525 PMC4775008

[26] Aishuwariya T, Ramesh S. An Update On Gutta-Percha Retrieval Methods. Int J Dent Oral Sci. 2021;8(2):1488–91. doi: 10.19070/2377-8075-21000302

[27] Trevisan L, Huerta IR, Michelon C, Bello MDC, Pillar R, Souza Bier CA. The Efficacy of Passive Ultrasonic Activation of Organic Solvents on Dissolving Two Root Canal Sealers. Iran Endod J. 2017;12(1):25–8. doi: 10.22037/iej.2017.05 28179919 PMC5282374

[28] Livada R, Hosn K, Shiloah J, Anderson KM. Management of heat-induced bone necrosis following thermal removal of gutta-percha. Quintessence Int. 2018;49(7):535–42. doi: 10.3290/j.qi.a40246 29662971

[29] Vijayalakshmi B, Sajjan G. An ex-vivo evaluation of thermal changes in periodontal ligament during the use of thermoplasticised gutta-percha obturating techniques. Int J Recent Sci Res. 2015;6(5):24–7.

[30] Zhou X, Chen Y, Wei X, Liu L, Zhang F, Shi Y, et al. Heat transfers to periodontal tissues and gutta-percha during thermoplasticized root canal obturation in a finite element analysis model. Oral Surg Oral Med Oral Pathol Oral Radiol Endod. 2010;110(2):257–63. doi: 10.1016/j.tripleo.2010.04.005 20659703

[31] Mircheska M, Stojanovska V, Redzep E, Popovska L. Evaluation of apical extrusion during conventional retreatment with three endodontic systems. Balkan J Dent Med. 2020;24(3):142–7. doi: 10.2478/bjdm-2020-0023

[32] Sayed N, Hussein S, Yehia T. Efficiency of four retreatment rotary file systems in removal of filling material from root canal (in vitro study ). Egyptian Dental Journal. 2023;69(4):3159–66. doi: 10.21608/edj.2023.213729.2575

[33] Ciftcioglu E, Sungur Guzel R, Akbal Dincer G, Karakaya G, Kucukay ES. Efficiency of ProTaper Universal Retreatment, Reciproc Blue and XP-endo Shaper in the removal of a bioceramic-based root canal filling. Eur Oral Res. 2023;57(3):159–64. doi: 10.26650/eor.20231081837 37929225 PMC10622151

[34] Boariu M, Nica L-M, Marinescu A, Ganea E, Velea O, Pop D. Efficiency of eucalyptol as organic solvent in removal of gutta-percha from root canal fillings. EV CHIM (Bucharest). 2015;66(6):907–10.

[35] Moreira CHC, Zanatta FB, Antoniazzi R, Meneguetti PC, Rösing CK. Criteria adopted by dentists to indicate the extraction of periodontally involved teeth. J Appl Oral Sci. 2007;15(5):437–41. doi: 10.1590/s1678-77572007000500012 19089175 PMC4327266

[36] Rossi-Fedele G, Ahmed HMA. Assessment of Root Canal Filling Removal Effectiveness Using Micro-computed Tomography: A Systematic Review. J Endod. 2017;43(4):520–6. doi: 10.1016/j.joen.2016.12.008 28214018

[37] Çiçek E, Koçak MM, Koçak S, Sağlam BC. Comparison of the amount of apical debris extrusion associated with different retreatment systems and supplementary file application during retreatment process. J Conserv Dent. 2016;19(4):351–4. doi: 10.4103/0972-0707.186456 27563185 PMC4979283

[38] Joseph M, Ahlawat J, Malhotra A, Rao M, Sharma A, Talwar S. In vitro evaluation of efficacy of different rotary instrument systems for gutta percha removal during root canal retreatment. J Clin Exp Dent. 2016;8(4):e355–60. doi: 10.4317/jced.52488 27703601 PMC5045680

[39] Giuliani V, Cocchetti R, Pagavino G. Efficacy of ProTaper universal retreatment files in removing filling materials during root canal retreatment. J Endod. 2008;34(11):1381–4. doi: 10.1016/j.joen.2008.08.002 18928852

[40] Wolcott JF, Himel VT, Hicks ML. Thermafil retreatment using a new “System B” technique or a solvent. J Endod. 1999;25(11):761–4. doi: 10.1016/S0099-2399(99)80127-7 10726547

[41] Abramovitz I, Relles-Bonar S, Baransi B, Kfir A. The effectiveness of a self-adjusting file to remove residual gutta-percha after retreatment with rotary files. Int Endod J. 2012;45(4):386–92. doi: 10.1111/j.1365-2591.2011.01988.x 22283664

[42] Das S, De Ida A, Das S, Nair V, Saha N, Chattopadhyay S. Comparative evaluation of three different rotary instrumentation systems for removal of gutta-percha from root canal during endodontic retreatment: An in vitro study. J Conserv Dent. 2017;20(5):311–6. doi: 10.4103/JCD.JCD_132_17 29386777 PMC5767824

[43] Betti LV, Bramante CM, de Moraes IG, Bernardineli N, Garcia RB. Comparison of GPX with or without solvent and hand files in removing filling materials from root canals—An ex vivo study. Oral Surg Oral Med Oral Pathol Oral Radiol Endod. 2010;110(5):675–80. doi: 10.1016/j.tripleo.2010.06.005 20955955

[44] Campello AF, Almeida BM, Franzoni MA, Alves FRF, Marceliano-Alves MF, Rôças IN, et al. Influence of solvent and a supplementary step with a finishing instrument on filling material removal from canals connected by an isthmus. Int Endod J. 2019;52(5):716–24. doi: 10.1111/iej.13047 30481389

[45] Kazi FM, Asghar S, Fahim M. Dissolving efficacy of different endodontic solvents for gutta percha with varying time intervals. J Pak Dent Assoc. 2018;27(3):110–4. doi: 10.25301/jpda.273.110

[46] Makkar S, Mushtaq U, Kaur T, Sharma M, Mushtaq F, Thakur D. Hypersensitivity reaction to orange oil gutta-percha solvent in dental office. Endodontology. 2021;33(2):107–11. doi: 10.4103/endo.endo_121_20

[47] Tambe VH, Nagmode PS, Vishwas JR, P SK, Angadi P, Ali FM. Evaluation of the Amount of Debris extruded apically by using Conv-entional Syringe, Endovac and Ultrasonic Irrigation Technique: An In Vitro Study. J Int Oral Health. 2013;5(3):63–6. 24155604 PMC3769864

[48] Kasam S, Mariswamy AB. Efficacy of Different Methods for Removing Root Canal Filling Material in Retreatment - An In-vitro Study. J Clin Diagn Res. 2016;10(6):ZC06-10. doi: 10.7860/JCDR/2016/17395.7904 27504397 PMC4963757

[49] Sainika S. Editorial on Passive Ultrasonic Irrigation and the Gentle-Wave System as Endodontic Retreatment Adjuvants. 2021;5:1. doi: 10.36648/2469-2980.21.7.E17

[50] Siraparapu KR, Moinuddin K, Behera R, Taduri V, Durgam H, Ramachandruni N. A Comparative Evaluation of the Dissolving Abilities of Eucalyptus, Orange, and Castor Oils in Endodontic Retreatment Using Conventional and Rotary Techniques. Cureus. 2024;16(7):e64063. doi: 10.7759/cureus.64063 39114210 PMC11304361

[51] Zhang Q, Gu J, Shen J, Ma M, Lv Y, Wei X. Apically extruded debris, canal transportation, and shaping ability of nickel-titanium instruments on contracted endodontic cavities in molar teeth. J Oral Sci. 2023;65(4):203–8. doi: 10.2334/josnusd.23-0050 37532528

[52] Khanvilkar U, Dhok V, Misra R, Pagariya G, Agarwal RS, Deshpande S. Comparative Evaluation of Debris Extrusion, Remaining Dentin Thickness and Fracture Resistance of Endodontically Treated Teeth Using Rotary and Reciprocating Endodontic File Systems: An In Vitro Study. Bangladesh J Med Sci. 2024;23(10):S87–91. doi: 10.3329/bjms.v23i10.71745 PMC10441161 37609092

[53] Ahmad MZ. Assessment of Debris Extrusion in Curved Canals: An In Vitro Analysis of Various Single‐File Endodontic Instrumentation Systems. Int J Dent. 2024;2024:8367693. doi: 10.1155/2024/8367693 39007058 PMC11245337

[54] Fiorillo L, D’Amico C, Meto A, Mehta V, Lo Giudice G, Cervino G. Sodium Hypochlorite Accidents in Endodontic Practice: Clinical Evidence and State of the Art. J Craniofac Surg. 2024;35(7):e636–45. doi: 10.1097/SCS.0000000000010407 39418527

